# Child’s Developmental Disabilities and Parental Well-being in Midlife and Old Age: Does Race Matter?

**DOI:** 10.1093/geroni/igaa057.3365

**Published:** 2020-12-16

**Authors:** Juha Lee, Manjing Gao, Chioun Lee

**Affiliations:** University of California, Riverside, Riverside, California, United States

## Abstract

Having a child with developmental disabilities (DD) compromises parents’ health and well-being. We have little knowledge on whether the association is robust to the presence of exposure-outcome confounders and how it varies by race. Guided by life-course perspectives, we evaluate (1) the association between having a child with DD and parental well-being and (2) racial disparities in the likelihood of having a child with DD (differential exposure), and/or the effect of having a child with DD on parental well-being (differential vulnerabilities). We advance prior studies by including a wide array of parent’s early-life adversities (ELAs, e.g., poverty and abuse), which may link the predictor to the outcome. Using the core, Refresher, and Milwaukee samples from Midlife in the United States (N=9,640, 25% non-Whites), we conducted regression analysis with race as a moderator. Compared to having a healthy child, parents having a child with DD reported lower well-being even after controlling for ELAs. While the likelihood of having a child with DD (around 10%) is similar for both non-Hispanic Whites and African Americans, African American parents are more adversely affected by having a child with DD across most of the eudaimonic well-being indicators (i.e., autonomy, self-acceptance, positive relationships with others, personal growth, environmental mastery). The later-life well-being of racial minorities is disproportionally affected by having a child with DD. Future research avenues include identifying life-course pathways that contribute to this differential vulnerability.

